# Perceptions of Infant Cry Sounds Among Tobacco and Cannabis Using Mothers and Their Association with Tobacco and Cannabis Cravings

**DOI:** 10.3390/children12081006

**Published:** 2025-07-31

**Authors:** Pamela Schuetze, Madison R. Kelm, Olivia Bell, Rina D. Eiden

**Affiliations:** 1 Department of Psychology, SUNY Buffalo State University, Buffalo, NY 14222, USA; opb5142@psu.edu; 2 The Pennsylvania State University, University Park, PA 16802, USA; mrk5763@psu.edu (M.R.K.); rina.eiden@psu.edu (R.D.E.)

**Keywords:** maternal depression, prenatal tobacco and cannabis co-exposure, maternal perceptions of infant cry sounds, maternal anger/hostility, tobacco and cannabis cravings

## Abstract

**Background/Objectives**: We examined maternal perceptions of infant cries as a mediator between maternal tobacco/cannabis use, psychological distress (depression/anger/hostility) and reported cravings for cigarettes and/or cannabis across two time points. **Methods**: A total of 96 substance-using mothers (35 tobacco-only and 61 tobacco/cannabis) were recruited in pregnancy. Maternal substance use and psychological distress were measured when their children were school age (5–6 years, T1). At the middle childhood assessment (9–12 years, T2), mothers listened to a standardized set of newborn cries and, afterwards, rated their aversiveness, impact on negative affect, and their tobacco/cannabis cravings. **Results**: Higher levels of maternal depressive symptoms at T1 were associated with perceptions of cries as being more aversive at T2, which, in turn, were associated with increased cannabis cravings at T2. At T1, higher depressive symptoms predicted increased tobacco cravings and higher maternal anger/hostility predicted increased cannabis cravings. **Conclusions:** Results highlight the role that infant cries and psychological distress play in cravings among tobacco/cannabis-using mothers.

## 1. Introduction

Tobacco and cannabis are two of the most commonly used drugs by young mothers and have important implications for parenting. Although rates of maternal tobacco use continue to decline [1], approximately 10% of adult women use tobacco. Conversely, rates of regular cannabis use among adults have climbed from about 7% in 2013 to 19% in 2021 [2] and the number of alternative forms of consumption [3] as well as the amount of tetrahydrocannabinol (THC) in cannabis has increased substantially over the past two decades [4,5]. Individuals who use tobacco often also use cannabis and the two substances are frequently smoked together [6,7,8]. Continued substance use is positively related to cravings and researchers have also documented associations between cravings and stress and negative affect [9,10,11]. However, it is not clear how common stressors related to parenting, such as infant crying, may be associated with cravings among mothers who use cannabis and/or tobacco.

### 1.1. Tobacco and Cannabis Cravings

Although the substantial literature has reported a positive association between negative affect and craving [11,12,13], these associations differ across substances and situations and are often not considered in the context of parenting which may elicit and/or exacerbate both negative affect and craving. For example, increased event negativity and symptoms of anxiety have been associated with tobacco cravings [10,14] and the association between tasks that elicit negative mood and tobacco cravings are stronger for women than for men [15]. Some studies have also suggested that the anticipation of relief from negative affect is associated with cannabis cravings [16]. However, after statistically controlling for initial levels of cravings, one study found that sad mood was not associated with tobacco cravings but was inversely associated with cannabis cravings [10]. The finding that positive mood was associated with cannabis cravings has been noted in studies using ecological momentary assessment that collects real-time data in the natural environments of substance using adults [17]. Thus, there are mixed findings with regard to the relation between cannabis craving and positive versus negative mood. In addition, many studies that have examined these constructs have utilized samples of individuals seeking treatment for addiction, making it unclear whether these findings generalize to nonclinical samples, to parenting contexts, or to mothers who use tobacco and/or cannabis. We examined associations between maternal tobacco/cannabis use, psychological distress, and tobacco/cannabis cravings in the context of exposure to infant distress signals (crying). Infant distress signals were used as an index of a typical situation frequently experienced by caregivers that may be perceived as aversive and stressful.

### 1.2. Maternal Tobacco and Cannabis Use and Maternal Negative Affect

Maternal substance use is a marker for a number of risk factors that may have implications for increased cravings such as maternal depression and anger/hostility. Both maternal smoking [18,19] and cannabis use [20,21] during pregnancy are consistently associated with higher levels of depression. Maternal tobacco and cannabis use are also associated with increased symptoms of anger/hostility. For example, mothers who smoke during pregnancy have higher levels of maternal anger/hostility across pregnancy, infancy, and toddlerhood [22] and mothers who smoked consistently across pregnancy had higher levels of anger/hostility and aggression than individuals who quit or reduced their tobacco use during pregnancy [23]. Similarly, studies of maternal tobacco and cannabis co-use found that women who co-used tobacco and cannabis experienced more anger/hostility during the prenatal period [24] and reported a smaller decrease in anger/hostility throughout infancy and toddlerhood [25].

### 1.3. Maternal Tobacco and Cannabis Use, Negative Affect and Responsiveness to Infant Signals

Infant distress signals are one particularly salient aspect of the parenting context that may explain, in part, pathways from maternal substance use and negative affect to cravings for cannabis and/or tobacco. Mothers who use substances are more likely to have difficulties recognizing infant emotional signals [26] and may display reduced maternal responsiveness and sensitivity [27], which may be due, in part, to overlapping neural circuits for addiction and parenting [28]. For example, mothers who used cocaine rated infant cry sounds as being less perceptually salient and less likely to elicit nurturant caregiving responses [29]. Similarly, polysubstance-using mothers have been found to have reduced neural activation in areas that are associated with reward and motivation as well as in areas responsible for cognitive control [28]. According to the reward-stress dysregulation model [30], maternal nicotine use can negatively impact the maternal reward and regulation system lessening the salience of infant signals which, in turn, leads to reduced maternal sensitivity to infant cues. Mothers who use tobacco have been shown to have delayed neural activation in response to infant faces supporting the idea that tobacco using mothers perceive infant cues as being less salient [30]. Although research on cannabis and parenting during the early years is quite limited, there is some evidence indicating that cannabis use is associated with maternal emotional dysregulation as well as alterations in the endocrine system that is related to caregiving [31,32]. Thus, it is possible that maternal cannabis use may be associated with altered perceptions of infant signals, and this was examined in the current study.

Maternal psychological distress is also associated with impairments in responsiveness to infant signals. Studies have shown that depressed women rate infant cry sounds as being less perceptually salient and less likely to prompt active, responsive caregiving responses [33] and that they engage in less active and sensitive caregiving in response to their crying infants [34]. Depressed mothers are also less responsive to changes in characteristics of infant cry sounds that have been shown to be critical indicators of an infant’s state [35]. It is not clear, however, how maternal psychological distress among substance-using women is associated with perceptions of infant distress signals or if altered perceptions of infant distress signals would lead to cravings for tobacco/cannabis. Thus, the purpose of this study was to test two primary aims. The first aim was to examine direct associations between the maternal amount of tobacco/cannabis use and psychological distress in early school age to cravings for tobacco and cannabis during middle childhood. The second aim was to examine whether maternal perceptions of infant distress signals may mediate these associations. In particular, based on existing research indicating that tobacco using mothers find cues to be less salient, we hypothesized that mothers who used tobacco would find infant cries to be less aversive and would be less likely to have increased cravings for tobacco. Furthermore, because maternal cannabis use disrupts maternal emotion regulation, we hypothesized that maternal cannabis use would be associated with increased negative affect and perceptions of infant cry sounds as being more aversive which, in turn, would be associated with increased cannabis cravings.

## 2. Materials and Methods

### 2.1. Participants

This sample consisted of 96 tobacco and tobacco-cannabis using women (*n* = 35 in the tobacco-using group and *n* = 61 in the tobacco and cannabis co-using group) recruited in the first trimester of pregnancy and participating in a larger longitudinal study of prenatal substance exposure. The current analyses focused on the assessments conducted at the following time points: (1) during pregnancy and after delivery, (2) when the children were at early school age (referred to as T1; *M_years_* = 5.74, *SD* = 0.51) and (3) during middle childhood (referred to as T2; *M_years_* = 10.05, *SD* = 0.85). Mothers were primarily young (*M_age_* = 24.41, *SD* = 5.32), low-income, women of color (58.33% Black, 8.33% Latinx, 5.21% more than one race) with low formal education, with 1–2 children (see Table 1).

### 2.2. Procedures

Mothers in the study provided informed consent at their first prenatal appointment and additional consents at the early school-age and middle childhood assessments (see Table 2 for a list of measures collected at each time point). Measures of maternal substance use and anger/hostility have been used in analyses reported in previous publications [36,37,38,39]. However, the data pertaining to the maternal perceptions of infant cry sounds and tobacco and cannabis cravings have not been previously published. As part of their participation in the ongoing, longitudinal study, mothers provided daily data on substance use using calendar-based interviews (Timeline Followback interview, TLFB, see below) at each of the prenatal assessments as well as at the middle childhood assessment. In addition, we assayed maternal saliva samples for metabolites of tobacco and cannabis during pregnancy (see [40,41]). We also collected infant meconium at birth which we assayed for tobacco and cannabis metabolites (see [41,42]). Together, with the self-reports of substance use during the prenatal assessments, we used the saliva and meconium assays to determine group status for pregnancy substance use which was then used to determine which mothers to include in the current analyses. At the early school age assessment, mothers rated their depressive symptoms and symptoms of anger/hostility. At the middle childhood assessment, mothers reported on their substance use using the TLFB and listened to a standardized set of representative newborn cries and rated the aversiveness of each cry as well as the impact of the cry on their negative affect (see below). In addition, mothers rated their tobacco and cannabis craving after listening to the cries. Mothers were paid for each pre and postnatal assessment on an escalating scale as an incentive to participate in the larger, ongoing longitudinal study.

### 2.3. Measures

#### 2.3.1. Depressive Symptoms

We measured depressive symptoms at the early school-age assessment with the Depression Scale of the Brief Symptoms Inventory (BSI) [43]. The BSI is a 53-item self-report that measures feelings of anger, hostility, and acts of aggression over the past 7 days. Respondents rated each item on a Likert-like scale from 1 (“Not at all”) to 5 (“Extremely”). In the present analyses, we used the depression subscale which had good internal reliability (Cronbach’s α = 0.86).

#### 2.3.2. Anger/Hostility

We measured symptoms of anger, hostility, and aggression at the early school-age assessment with the Buss–Perry Aggression Questionnaire (BPAQ) [44]. The BPAQ is a 29-item self-report that measures feelings of anger, hostility, and acts of aggression. Respondents rate each item on a Likert-like scale from 1 (“Extremely uncharacteristic of me”) to 5 (“Extremely characteristic of me”). The BPAQ consists of four subscales including anger, hostility, physical, and verbal aggression and also includes a total score with higher scores indicating more anger, hostility, and aggression. In the present analyses, we used the total score which had excellent internal reliability (Cronbach’s α = 0.92).

#### 2.3.3. Substance Use

We used multiple methods including self-report and biological assays to assess maternal substance use during pregnancy. To assess self-reported substance use, we used the Timeline Follow-Back Interview (TLFB; [45]), a reliable calendar-based interview and valid method of determining individual patterns of substance use, including tobacco and cannabis [46]. The TLFB has good test–retest reliability and has been shown to be strongly correlated with other intensive self-report measures of substance use [47]. At the end of each trimester, we provided mothers with a calendar on which they identified the approximate date of conception and salient events in each month (e.g., holidays, birthdays, parties, sports events, anniversaries, funerals, vacations, etc.) as anchor points to help them recall their substance use. The TLFB yielded daily data on maternal tobacco and cannabis use across each trimester of pregnancy. In addition, we assessed prenatal substance use via both maternal and infant biomarkers. Maternal oral fluid samples were collected during pregnancy and infant meconium at birth. Both types of samples were assayed for cotinine, and meconium was also assayed for tetrahydrocannabinol (THC) and metabolites (see [40,41,42]). Postnatally, we assigned mothers to two mutually exclusive groups: the tobacco and cannabis co-use group or the tobacco-only group utilizing both self-report and biomarkers. We assigned mothers to the tobacco-cannabis co-use group if they self-reported using tobacco and/or cannabis on the TLFB during the prenatal or middle childhood assessments, if maternal prenatal oral fluid samples were positive for cotinine, and/or if infant meconium was positive for metabolites of nicotine or cannabis (e.g., cotinine, nicotine, trans-3′ hydroxycotinine, or THC and metabolites). We assigned mothers to the tobacco-only group if they self-reported using tobacco on the TLFB during the prenatal or middle childhood assessments, if maternal prenatal oral fluid samples were positive for cotinine, and/or if infant meconium was positive for cotinine and did not find evidence of cannabis use by either self-report or biological assay.

At the middle childhood visit, mothers reported on their tobacco and cannabis using the TLFB as described above. We asked mothers to report on their tobacco and cannabis use since the prior laboratory assessment which occurred when children were early school-aged. Only mothers who were prenatally assigned to the tobacco-only group or the tobacco-cannabis co-use group or who used tobacco and/or cannabis in middle childhood were included in the current analyses. Using the TLFB, we derived the average number of cigarettes smoked per day and the average number of joints per day in middle childhood which we used in the current analyses.

#### 2.3.4. Maternal Perceptions of Infant Cries

To assess maternal perceptions of infant cries, we created two composite variables. The first variable reflected how aversive mothers perceived the infant cries to be. We assessed this construct by having mothers listen to three standard infant cries and then rating three items assessing how unpleasant, urgent, and irritating mothers perceived the cry to be. Mothers rated cry aversiveness on a scale from 1 “Not unpleasant at all” to 5 “Extremely unpleasant.” The cry aversiveness variable had adequate internal reliability (Cronbach’s α = 0.68). Next, we created a second composite variable reflecting the impact of infant cries on maternal negative affect. After listening to the three standard infant cries, mothers rated three additional items assessing how sad, anxious, and angry they felt. Mothers rated the impact of the cry on a scale from 1 “Not sad at all” to 5 “Extremely sad.” The cry impact variable had adequate internal reliability (Cronbach’s α = 0.74).

#### 2.3.5. Tobacco and Cannabis Craving

After listening to the infant cries, mothers rated their cannabis and tobacco cravings using four items taken from the Questionnaire of Smoking Urges-4 (QSU-4) [48]. Mothers rated each of the four items for cannabis and tobacco separately. Mothers rated their cravings on a scale from 0 “Do not agree” to 100 “Strongly agree” with 50 “Moderately agree” as the anchor point. The composite tobacco and cannabis craving variables had excellent internal reliabilities, (Cronbach’s α = 0.97 and 0.91), respectively.

### 2.4. Missing Data

Overall, 2.1% of the data was missing for the mediating (cry aversiveness and the impact of infant cries on maternal negative affect) and endogenous variables (tobacco and cannabis craving) measured at the middle childhood assessment. Mothers who had missing data on mediating variables also had missing data on endogenous variables. Overall, 85 mothers had complete data. We used independent samples *t*-tests and chi-square tests of independence to assess whether mothers with missing versus complete data differed on demographic and primary study variables. Mothers with complete versus incomplete data did not significantly differ on education, parity, child sex, or other demographic variables. However, mothers with complete versus missing data for tobacco and cannabis craving and cry perception variables differed on maternal age, *t*(94) = 2.36, *p* = 0.02, such that older mothers were more likely to have missing data. There was no association between missingness and maternal average number of cigarettes or joints at the middle childhood visit or maternal anger/hostility at the early childhood visit (*p* > 0.10). However, mothers with complete versus missing data for tobacco and cannabis craving and cry perception variables differed on maternal depressive symptoms, *t*(86) = −6.01, *p* < 0.001, such that mothers with fewer depressive symptoms were more likely to have missing data. Thus, data met criteria for missing at random [49], maternal age was included in models as a covariate, and we handled missing data using full information maximum likelihood [50], resulting in an analytic sample of 96.

### 2.5. Analytic Plan

**Figure 1:**
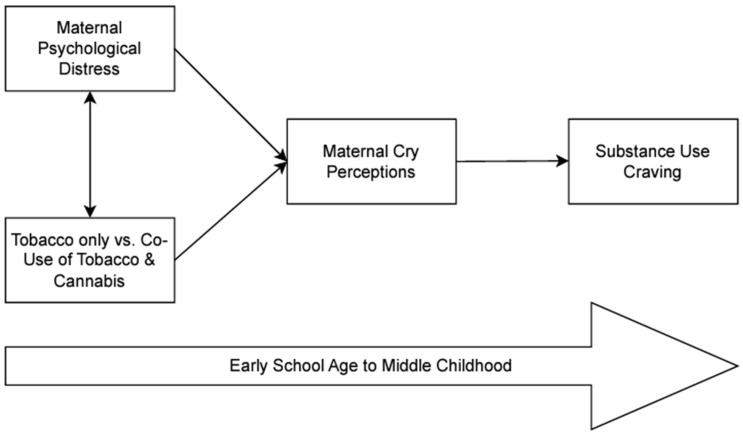
Conceptual model.

We used a path analytic model to test both of our specific aims by testing the fit of the conceptual model (see Figure 1) using Mplus, Version 8 software [51]. To assess potential mediation, we tested indirect effects using both the bias-corrected and percentile bootstrap methods [52,53]. We used 5000 bootstrap samples and determined whether indirect effects were significant using 95% confidence intervals (CIs). We assessed overall model fit using the chi-square difference test, root mean square error of approximation (RMSEA), standardized root mean square residual (SRMR), and comparative fit index (CFI).

## 3. Results

### 3.1. Preliminary Analyses

Descriptive statistics are presented in Table 2. Correlations between study variables included in the model are presented in Table 3.

### 3.2. Primary Analyses

**Figure 2:**
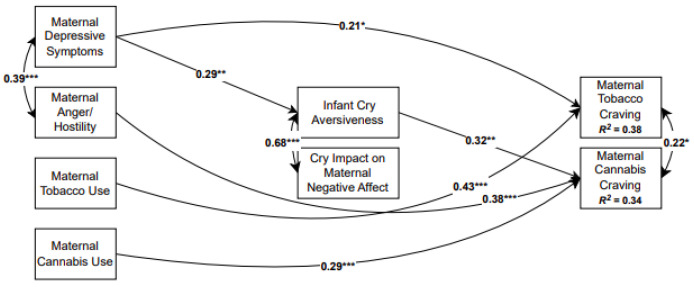
Final model note. Numbers represent standardized path coefficients; nonsignificant paths are not depicted for ease of presentation. Covariates included maternal age at recruitment and number of live births. * *p* < 0.05, ** *p* < 0.01, *** *p* < 0.001.

The analytic model included maternal psychological distress at early school-age (maternal depressive symptoms, maternal anger/hostility) and the average number of cigarettes and joints per day at the middle childhood visit as exogenous variables. To test our first aim, we modeled causal paths from these variables to maternal cravings for tobacco and cannabis use. To test our second, mediational aim, we modeled causal paths from maternal substance use and psychological distress to maternal perceptions of infant cry aversiveness and the impact of infant cries on maternal negative affect (see Figure 2). Finally, we included paths from the two maternal perception of infant cry variables to maternal tobacco and cannabis craving at middle childhood and within-time covariances for all variables. As described above, maternal age at recruitment was included as a covariate because of its association with missing data for substance use and cry perceptions and because it was associated with cigarette craving. In addition, we included parity as a covariate because, conceptually, it is likely to be associated with how mothers perceive cry sound and because it was associated with maternal negative affect after hearing infant cry sounds.

### 3.3. Overall Model

Path analytic model results are presented in Figure 2. The direct effects are summarized in Table 4 and the indirect effects are summarized in Table 5. The hypothesized model tested whether maternal perceptions of infant cries mediated the relation between maternal psychological distress and substance use variables and tobacco and cannabis craving (aim 2). This indirect effects model was not a good fit to the data. In the next step, we added theoretically justified direct paths from the exogenous variables (maternal average tobacco and cannabis use and psychological distress) to the two craving variables sequentially and retained significant direct associations (aim 1). Model fit indices indicated that this model had an excellent fit to the data, *χ^2^* (18) = 21.01, *p* = 0.28; *RMSEA* = 0.04, 90% CI [0.00, 0.10]; *CFI* = 0.98 *SRMR* = 0.05. Higher levels of maternal depressive symptoms at early school-age were prospectively associated with maternal perception of infant cries as more aversive, (*β* = 0.29, *p* = 0.009) and this, in turn, was associated with greater cannabis craving, (*β* = 0.32, *p* = 0.006). In addition, higher levels of maternal depressive symptoms at early school-age and higher average daily cigarette use at middle childhood directly predicted more tobacco craving, (*β* = 0.21, *p* = 0.021) and (*β* = 0.43, *p* < 0.001), respectively. Finally, higher levels of maternal anger/hostility at early school-age and higher average daily cannabis use at middle childhood predicted greater cannabis craving, (*β* = 0.38, *p* < 0.001) and (*β* = 0.29, *p* < 0.001), respectively. Overall, the model accounted for 37.8% of the variance in maternal tobacco craving and 33.7% of the variance in maternal cannabis craving. When tested using the bias-corrected method, the indirect association between maternal depressive symptoms and cannabis craving via cry aversiveness was significant, *β* = 0.09, 95% CI [0.02, 0.23]. However, given recent evidence suggesting that bias-corrected bootstrapping may inflate type I error [53], we also tested indirect effects using the percentile bootstrap confidence interval (which does not adjust for bias). Using this method, we found that the confidence interval for the indirect effect of maternal depressive symptoms on cannabis craving via cry aversiveness included 0 and was thus, not significant, *β* = 0.09, 95% CI −0.00, 0.19. Conservatively, we chose not to interpret this indirect effect.

## 4. Discussion

The purpose of this study was to examine a model hypothesizing direct and indirect linkages from maternal tobacco and cannabis use and psychological distress to cravings for tobacco and cannabis. First, consistent with hypotheses, we did find that maternal tobacco and cannabis use when the child was in middle childhood were positively associated with reported tobacco and cannabis cravings, respectively, after hearing infant cry sounds. This finding is similar to previous research that has found an increase in substance use cognitions and behaviors in response to infant crying [54] and is consistent with the reward-stress dysregulation model of parenting and addiction that posits that increased stress in response to infant distress signals and other affective cues may lead to increased cravings for substances [30]. However, substance use was not associated with maternal ratings of the cry sounds. Previous work has shown impairments in autonomic regulation among tobacco and cannabis users [55] and in maternal responsivity to infant cry sounds [56]. Furthermore, dysregulated processes have been associated with increased substance cravings [57]. Thus, although hearing infant cries may not impact cognitive perceptions of cries, there may be physiological impacts that result in increased cravings for tobacco and cannabis. Future research should explore physiological responsiveness as a possible mechanism that explains increased cravings in response to infant distress signals.

We also found that symptoms of both maternal depression and anger/hostility were associated with cravings. First, consistent with previous studies [10,14,15], we found that symptoms of depression were associated with increased cravings for tobacco. These findings add to a robust literature linking depression with an increased likelihood for using cigarettes and other tobacco products and with increased daily use of tobacco products [58,59,60,61]. Given the intergenerational impact of maternal cigarette smoking, treatment programs for mothers who smoke cigarettes should incorporate interventions for depression, particularly in the context of parenting demands. Second, we found that symptoms of anger/hostility were associated with increased cravings for cannabis. These findings are consistent with previous work that has shown a positive association between cannabis use and anger/hostility [62,63,64,65] and that have shown that cannabis use leads to decreases in anger/hostility [65]. Our work, however, extends these findings to a sample of mothers who are not seeking treatment for their tobacco and/or cannabis use. Our findings also lend further support to the importance of considering substance type when exploring predictors of cravings and highlight the importance of considering the role that affective psychopathology has for cravings in the context of parenting.

Furthermore, although we know quite a bit about the impact of child behaviors and characteristics on parental mental health and behaviors (evocative child effects), less is known about how caregivers perceive challenging behaviors and how that might impact continued substance use. Therefore, we were also interested in exploring whether maternal perceptions of infant distress signals mediate any associations between maternal psychological distress (depression, anger and hostility), tobacco and cannabis use and reported cravings for cigarettes and/or cannabis. Although we did not find that maternal perceptions of infant cry aversiveness mediated associations between maternal psychological distress or substance use and cravings, higher levels of maternal depressive symptoms were associated with perceptions of infant cry sounds as being more aversive. We also found associations between perceptions of cry aversiveness and increased cravings for cannabis. Thus, although perceptions of cry aversiveness were not a significant mediator, they were a direct predictor of cannabis cravings. Although previous research found that severely depressed women rated cries as being less perceptually salient [29,35] and exhibit reduced physiological and neurological responsivity to cry sounds [66,67] our findings are consistent with other research indicating that mothers who were more depressed reported perceiving their infant as more difficult [35] and may have more negative parent–infant interactions and reduced pleasure in maternal responsivity to infant cues [68,69]. Furthermore, maternal perceptions of challenging child behaviors such as crying and sleep, have implications for maternal mental health and mother–child bonding [70]. Understanding the perceptions that mothers have of child behaviors can inform interventions by allowing clinicians to effectively allocate resources to the mothers that can most benefit.

We also found that the paths from maternal tobacco and cannabis use were not associated with perceptions of infant cry sounds, findings that are not consistent with others indicating that tobacco using mothers find infant cues to be less salient [30]. It is important to note that other research that has examined associations between maternal depression and responsivity to infant cry sounds has not been conducted within the context of substance use and has focused on perceptions of women during the immediate postpartum period. We assessed maternal perceptions of infant cry sounds when their child was early school-age rather than when their child was an infant. Thus, infant distress signals may not be as salient at this point in their parenting trajectory.

Despite numerous strengths of the study, including the prospective, longitudinal nature of the study and the multi-method assessments of substance exposure, several limitations should be noted. First, our measure of maternal cravings was based solely on maternal self-reports. Although our measure of responsivity to infant cry sounds was also based on maternal self-report, understanding how parents perceive challenging behaviors in their children are important for understanding how to best target interventions to help mother-child dyads. In addition to obtaining maternal self-reports of their perceptions, future research should consider including measures of physiological responsivity to assess the impact that infant distress signals and/or other child behaviors have on maternal stress responsivity. Second, future studies should also examine maternal perceptions to infant cry sounds among tobacco and cannabis using mothers during the early postpartum period when this is a particularly salient cue that may affect caregiving behaviors. Future studies could also consider using child signals from the mother’s actual child to increase the ecological validity of these findings. Furthermore, because infant temperament and emotion regulation are associated with maternal mental health and parenting behaviors, future research should explore the association between infant behaviors and characteristics and maternal cravings for substances. Another limitation of our study is that it is unclear if increased cravings would be found in other parenting contexts and whether any increased cravings during parenting stressors would translate into continued substance use. Future studies should explore predictors of cravings among parents and longitudinally assess how any such cravings impact ongoing substance use. A final limitation is that our sample size fell just short of recommendations for a minimum samples size of 100 for path analysis [71] although this recommendation is based on the finding that many models fail to converge with smaller sample sizes.

Despite these limitations, this study highlights the role that infant distress signals play in cravings among mothers who use tobacco and cannabis. These associations are particularly important to understand since maternal tobacco and cannabis use is associated with higher levels of infant irritability and emotional and physiological dysregulation [26,27,38,39]. Given that infants are reliant on their caregivers for help with regulating their emotions during periods of distress [72] and that sensitive maternal responses to infant cues are important for healthy infant socioemotional development and for promoting optimal mother–infant relationships [73,74]. Greater awareness of the impact of infant crying on caregiver cravings for substances is important for the development of more effective interventions and will allow for more targeted support and advice as needed by substance using mothers.

## Figures and Tables

**Table 1:** Descriptive Statistics for Study Variables and Covariates.

	Tobacco	Co-Exposed	*F/χ*^2^ (*p*)	Partial η^2^
Maternal Substance Use				
Cigarettes Per Day	6.09 (5.89)	7.11 (5.86)	0.64 (0.43)	0.03
Joints Per Day	0 (0)	2.05 (3.92)	7.89 (0.006)	0.18
Maternal Psychological Distress				
BPAQ Total Score (Anger/Hostility)	2.29 (0.61)	2.52 (0.75)	3.50 (0.07)	0.14
BSI Subscale Score (Depressive Symptoms)	0.57 (0.71)	0.65 (0.72)	0.27 (0.61)	0.06
Maternal Cry Perception				
Impact on Negative Affect	2.98 (0.98)	2.89 (1.09)	0.13 (0.73)	0.04
Cry Aversiveness	3.23 (0.89)	3.37 (1.02)	0.41 (0.52)	0.05
Craving Scale Score				
Tobacco	0.67 (1.59)	0.58 (3.08)	0.13 (0.72)	0.01
Cannabis	0 (0)	0.33 (1.18)	10.12 (0.002)	0.16
Demographics				
Maternal Age	24.94 (5.56)	24.14 (5.22)	0.48 (0.49)	0.04
Maternal Education (years)	12.21 (1.86)	12.23 (1.9)	0.77 (0.58)	0.03
Race (% nonWhite)	59.28%	68.04%	9.64 (0.003)	

Note. BSI = Brief Symptom Inventory; BPAQ = Buss-Perry Aggression Questionnaire.

**Table 2:** Study timeline and sample size.

Assessment Point and Construct	Sample Size
Prenatal/Birth	
Maternal Substance Use–Self-report	*n* = 96
Salivary Assays for Substance Use	*n* = 96
Infant Meconium	*n* = 76
Early School-age–Kindergarten	
Maternal Depression	*n* = 91
Maternal Anger/Hostility	*n* = 89
Middle Childhood	
Maternal Tobacco/Cannabis Use	*n* = 96
Maternal Cry Perceptions	*n* = 94
Maternal Cravings for Tobacco/Cannabis	*n* = 94

**Table 3:** Bivariate correlations among primary study variables.

	1	2	3	4	5	6
1. Co-Use vs. Tobacco Use	-					
2. Maternal BPAQ Total Score		-				
3. Maternal Depression		0.39 ***	-			
4. Cry Impact on Negative Affect		0.07	0.19	-		
5. Cry Aversiveness		0.13	0.28 **	0.69 ***	-	
6. Tobacco Craving		0.42 ***	0.23 *	0.01	20	-
7. Cannabis Craving		−0.03	0.35 ***	0.16	0.20	0.20 *

* *p* = 0.05, ** *p* = 0.01, *** *p* < 0.001.

**Table 4:** Standardized direct effects in the path analysis model.

Direct Pathway	Direct Effect
Maternal Depressive Symptoms **→** Tobacco Craving	0.21 *
Maternal Tobacco Use **→** Tobacco Craving	0.43 **
Maternal Anger/ **→** Cannabis Craving	0.38 **
Maternal Cannabis Use **→** Cannabis Craving	0.29 **
Maternal Depressive Symptoms **→** Infant Cry Aversiveness	0.29 **
Maternal Anger/Hostility **→** Infant Cry Aversiveness	0.03
Maternal Tobacco Use **→** Infant Cry Aversiveness	−0.07
Maternal Cannabis Use **→** Infant Cry Aversiveness	−0.08
Maternal Depressive Symptoms **→** Cry Impact on Maternal Negative Affect	0.19
Maternal Anger/Hostility **→** Cry Impact on Maternal Negative Affect	−0.02
Maternal Tobacco Use **→** Cry Impact on Maternal Negative Affect	−0.05
Maternal Cannabis Use **→** Cry Impact on Maternal Negative Affect	−0.03

Notes: * *p* < 0.05. ** *p* < 0.01.

**Table 5:** Standardized indirect effects in the path analysis model.

Indirect Path	Lower 5%	Estimate	Upper 5%
Maternal Depressive Symptoms → Infant Cry Aversiveness → Maternal Tobacco Craving	−0.004	0.047	0.125
Maternal Depressive Symptoms → Cry Impact on Maternal Negative Affect → Maternal Tobacco Craving	−0.044	0.001	0.034
Maternal Depressive Symptoms → Infant Cry Aversiveness → Maternal Cannabis Craving	−0.001	0.091	0.190
Maternal Depressive Symptoms → Cry Impact on Maternal Negative Affect → Maternal Cannabis Craving	−0.129	−0.042	0.019

Notes. These values are the percentile based indirect effects.

## Data Availability

The raw data supporting the conclusions of this article will be made available by the authors on request.

## Abbreviations

The following abbreviations are used in this manuscript:

TLFB	Timeline Followback Interview
BSI	Brief Symptom Inventory
BPAQ	Buss-Perry Aggression Questionnaire
QSU-4	Questionnaire of Smoking Urges-4
RMSEA	Root mean square error or approximation
SRMR	Standardized root mean square residual
CFI	Comparative fit index
